# Antioxidant and Anti-Inflammatory Properties of Plants Extract

**DOI:** 10.3390/antiox8110549

**Published:** 2019-11-14

**Authors:** Mario Allegra

**Affiliations:** Department of Biological, Chemical and Pharmaceutical Sciences and Technologies, Università di Palermo, Via Archirafi, 28, 90123 Palermo, Italy; mario.allegra@unipa.it; Tel.: +39-091-238-96803

Inflammation is an adaptive response triggered by noxious stimuli and conditions such as infection and tissue injury [1,2]. It has extensively been demonstrated that strong and complex interconnections occur between oxidative stress and the inflammatory response [3]. Alterations of the endocellular redox state play a key role in the activation and/or dysfunction of immune cells. Along these lines, the plant kingdom contains an immense variety of secondary metabolites, named phytochemicals (PhC), with significant redox-modulating properties that have been recently shown to effectively modulate the inflammatory response [4]. First considered ‘health promoting’ by virtue of their radical-scavenging activity and/or direct antioxidant effects on cellular biomolecules [5], such compounds are now believed to interfere with cell functions by intercepting reactive species at the level of critical cell signalling pathways. In addition, knowledge of the interaction of these molecules with enzymes, receptors, and transcription factors has recently emerged [6,7].

Whilst it is crucial to identify, in a selected plant, the PhC responsible for the pharmacological effects, it is also important to analyse whether different PhC, within a plant extract, synergistically cooperate to exert the specific health benefit. Along these lines, this Special Issue has been devoted to collecting original research papers and reviews on the effects and mechanisms through which plants extracts exert anti-oxidative and anti-inflammatory effects. New information has been added in this field by means of 15 articles, with 14 original papers and one review.

In the first paper, Attanzio et al. report for the first time the polyphenol composition of a hydro-alcoholic extract of the sap from *Fraxinus angustifolia* Vahl, the so-called manna, and its reductive and antioxidant capacity in water, as well as in the lipid phase. Authors found that the extract was effective in scavenging several reactive radicals, including those derived from heme-proteins, showed the lipoperoxyl radical-scavenging capacity in solution, and, more importantly, provided antioxidant protection in a complex environment, such as human red blood cells. Interestingly enough, the extract also showed anti-inflammatory effects in an in vitro model of intestinal bowel disease [8].

In line with the current growing interest in cosmeceuticals, the work of Zovko Koncic et al. has optimized the extraction methods of phenolic compounds from licorice root using glycerol, a non-toxic and eco-friendly solvent. The prepared extracts displayed relevant and interesting radical-scavenging, antioxidant, and anti-inflammatory effects. These activities, together with the tyrosinase and elastase inhibitory activities, may suggest that these extracts are highly-active components for cosmeceuticals products with anti-aging properties [9].

In light of the negligible current knowledge about the chemical constituents and biological activity in the *Laserpitium* species, the work of Szewczyk et al. has undertaken the qualitative and quantitative analysis of phenolic acids and flavonoids in the etheric and methanolic extracts of the fruits of *Laserpitium kraffii* Crantz and evaluated their antioxidative and cytotoxic effects. The study led to the identification of 12 phenolic acids and nine flavonoids and showed that both extracts have high cytotoxic and antioxidant potential [10]. 

It is well known how the composition of a plant extract can vary considerably according to several factors, including the conservation procedure. In this regard, it appears that the work by Gabbianelli et al. on *Nigella sativa* oil obtained from a cultivar produced in the region Marche is of particular interest. The authors demonstrated that its thymoquinone content is higher than that from other cultivars in the Mediterranean area, Asia, and Indonesia. More interestingly, they showed how storage may affect thymoquinone content as well as the anti-inflammatory and antioxidant effects of the oil. Finally, they also demonstrated that both the antioxidant and anti-inflammatory effects of the oil does not simply rely on thymoquinone content, but also on other oil components that may exert synergistic effects [11].

A novel chemical investigation came from the work of Kukula-Koch et al. on the flavonoid constituents of *Crataegus almaatensis* leaves. The authors found that the three main flavonoids (hyperoside, quercitrin, and afzelin) optimized their isolation technique and evaluated extracts for their vascular, anti-nociceptive, and anti-inflammatory effects, in vitro and in vivo [12]. 

In light of the current interest on the alternative use of vegetal wastes, the study from Catoi et al. investigated 10 tomato varieties in terms of carotenoids content, phenolic composition, and their related antioxidant and antimicrobial activities. Taking into account the results obtained, the authors concluded that tomato industrial by-products may represent a source of natural bioactive molecules with applicability in the nutraceuticals and food industries [13]. 

Notwithstanding its nutritional role and properties, meat has been recently linked to an increased incidence of non-communicable diseases, such as colorectal cancer. In the study from Nieto et al., authors aimed to evaluate the inflammatory role of processed meat and develop a functional meat product enriched in natural extracts with antioxidant and anti-inflammatory properties. The authors found that cooked ham that is low in fat and salt had antioxidant and anti-inflammatory properties related to the inhibition of substances involved in gut inflammation, such as reactive oxygen species, nitric oxide, and cytokines produced by macrophages. These health-promoting inhibitory properties were significantly increased when the cooked ham was enriched with bioactive phytochemicals. Therefore, the authors proposed that incorporating botanical extracts rich in phenolic compounds (chlorogenic acid, catechins, rosmarinic acid, and hydroxytyrosol) in cooked ham is a good strategy to produce a healthy functional meat product [14].

Studies on the health-promoting effects of green tea have typically focused on catechins. Unlike catechins, green tea flavonols have received little attention with respect to their usage and functionality. In their paper, Kim et al. investigated the antioxidative, anti-inflammatory, and antiproliferative effects of flavonol glycoside (FLG)- and flavonol aglycone (FLA)-enriched fractions isolated from green tea extract. These fractions contained 16 and 13 derivatives, respectively, including apigenin, kaempferol, myricetin, and quercetin. FLA exhibited higher radical-scavenging activity than that of FLG. Interestingly, both attenuated intracellular oxidative stress in neuron-like PC-12 cells significantly reduced inflammatory genes in murine macrophages and inhibited proliferation of both the colon adenoma cell line DLD-1 and the breast cancer cell line E0771. Moreover, treatment with FLG or FLA combined with paclitaxel had synergistic anticancer effects on the DLD-1 cell line. The authors concluded that flavonols from green tea may exert beneficial health effects that may be superior to those of flavan-3-ols [15].

In light of the, so far, underexplored properties of *Salvia apiana* and *Salvia farinacea*, particular interest comes from the work by Cardoso et al., which demonstrates significant antioxidative and anti-inflammatory effects of these two *Salvia* extracts, which also exert cytotoxic and antimicrobial effects. 

In this regard, the authors suggest that the bioactive properties of the two extracts are associated with their phenolic components and, in the particular case of *Salvia apiana*, to its richness in rosmanol, hydroxycarnosic acid, and a derivative of sageone [16].

The accumulation and concentration of phytochemicals in different parts and organs of plants can considerably vary with regard to the function of these compounds in a plant’s lifecycle and growth phase. The work of Maslennikov et al. has investigated the accumulation of phenolic compounds and the antioxidant activity of extracts from different parts of *Rumex crispus* and *Rumex obtusifolius* (roots, stems, leaves, and reproductive organs), collected at both the flowering and fruiting stages. The maximum number of phytochemicals was found in the reproductive organs, both in the flowering and fruiting period. On the contrary, stems showed a minimum content of active compounds [17]. 

The valorisation of agrochemical wastes has been widely recognized as a crucial way to reduce the health spending and improve the accessibility of bioactive natural compounds. In line with this, Aiello et al. have investigated the chemical composition of three *Glycyrrhiza glabra* leaf extracts, as well as the anti-oxidative and anti-inflammatory profile of their three main components, i.e., pinocembrin, glabranin, and licoflavanone. Interestingly, the authors demonstrated a modulation of NF-κB/MAPK pathways by licoflavanone, highlighting the potential of this natural compound as a new scaffold in anti-inflammatory drug research [18].

Hyperglycemia-induced oxidative stress fosters severe vascular damage and induces an inflammatory state strictly implicated in the etiopathogenesis of atherosclerosis. In the work by Kim et al., *Carpinus turczaninowii* extract has been demonstrated to attenuate high glucose-induced inflammation and arterial damage. Relevantly, authors provided mechanistic insights of the protective effects observed, as well as the identification of 15 types of phenolic compounds present in the extract (including quercetin, myricitrin, and ellagic acid) that exhibit antioxidant and anti-inflammatory properties [19].

Anji white tea is a unique variety of green tea rich in polyphenols. In the work by Zhao et al., the effect of Anji white tea polyphenols on the prevention of carbon tetrachloride-induced liver injury was investigated. Interestingly, the extract effectively counteracted liver injury through a redox-, anti-inflammatory-, NF-κB-dependent mechanism with an efficacy comparable to that of silymarin [20].

In light of the previously reported data on the antioxidant and anti-inflammatory properties of a crude oil extracted from the seeds of *Nigella sativa* produced in the Marche region, this second work from Gabbianelli et al. has evaluated its analgesic and anti-inflammatory potential in a rat, adjuvant-induced arthritis model. Interestingly, at the same time, the authors evaluated its safety profile in vivo. Results showed that the extract was effective in the control of acute phase inflammation and prevented the development of rheumatoid arthritis (RA). On the other hand, it was not able to ameliorate the severity of arthritis in the chronic phase. Interestingly, despite the positive effects on the clinical signs of RA, the extract did not reduce plasma IL-6 levels, whereas it decreased plasma total cholesterol levels, strongly suggesting a potential control of cholesterol metabolism [21].

Finally, in their work, Kaliora and Papada reviewed the current knowledge on Mastiha, a natural product of the Mediterranean area found as a dried resinous exudate from the stems and branches of the tree, *Pistacia lentiscus*. A total of 19 studies were analyzed showing how, from preclinical evidence, the antioxidant potential of Mastiha may be correlated to the inhibition of protein kinase C, while its anti-inflammatory capacity is correlated to the inhibition of NF-κB activation [22].
